# REVA as A Well-curated Database for Human Expression-modulating Variants

**DOI:** 10.1016/j.gpb.2021.06.001

**Published:** 2021-07-03

**Authors:** Yu Wang, Fang-Yuan Shi, Yu Liang, Ge Gao

**Affiliations:** 1 State Key Laboratory of Protein and Plant Gene Research, School of Life Sciences, Biomedical Pioneering Innovation Center (BIOPIC) & Beijing Advanced Innovation Center for Genomics (ICG), Center for Bioinformatics (CBI), Peking University, Beijing 100871, China; 2 Human Aging Research Institute, School of Life Sciences, Nanchang University, Nanchang 330031, China

**Keywords:** Noncoding variant, Expression-modulating variant, Massively parallel reporter assay, Database, Benchmark

## Abstract

More than 90% of disease- and trait-associated human variants are noncoding. By systematically screening multiple large-scale studies, we compiled REVA, a manually curated **database** for over 11.8 million experimentally tested **noncoding variants** with expression-modulating potentials. We provided 2424 functional annotations that could be used to pinpoint the plausible regulatory mechanism of these variants. We further benchmarked multiple state-of-the-art computational tools and found that their limited sensitivity remains a serious challenge for effective large-scale analysis. REVA provides high-quality experimentally tested **expression-modulating variants** with extensive functional annotations, which will be useful for users in the noncoding variant community. REVA is freely available at http://reva.gao-lab.org.

## Introduction

Noncoding regions occupy the majority of the human genome [1]. It has been demonstrated that noncoding variants can affect the regulation of genes [2], and more than 90% of disease- and trait-associated variants are noncoding variants [3]. Noncoding variants that could affect gene expression can be considered as expression-modulating variants [4]. Several experimental assays have been developed to characterize expression-modulating variants. Genome editing technologies such as transcription activator-like effector nucleases (TALENs), zinc finger nucleases (ZFNs), and clustered regularly interspaced short palindromic repeats with Cas9 nuclease (CRISPR/Cas9) provide high-quality validated data but are generally low throughput [5], [6], [7]. Recently developed massively parallel reporter assays (MPRAs) can identify transcriptional regulatory elements in an efficient way, allowing systematic screening of tens of thousands of genetic variants for pinpointing the causal variants of complex traits [4], [8], [9]. All expression-modulating variants stored in MaveDB [10] are validated by the MPRA experiments. MPRAs have generated over 10 million human expression-modulating variants [11]; however, only around 30 thousand of them have been collected by MaveDB without any functional annotation, which hinders the further utilization of these data.

Although experimental assays for characterizing noncoding expression-modulating variants have generated a huge amount of data, it is still inadequate for covering all noncoding variants identified in human genomes. Therefore, multiple computational tools have been developed for identifying expression-modulating variants (Table 1). Transcription factors (TFs) could regulate genes through binding to sequence motifs [12], and noncoding variants could affect gene regulation by changing motifs [13]. FunSeq2 integrated a module for detecting motif-breaking and -gain events through the change of position weight matrix (PWM) and other functional annotations to prioritize cancer driver mutations [14]. Methods based on machine learning have been used wildly in biological researches [15]. CADD [16] used support vector machine (SVM) to classify variants into functional and nonfunctional variants, and GWAVA [17] used random forest to predict disease-related variants. Both CADD and GWAVA were based on supervised learning methods, while Eigen [18] implemented unsupervised learning methods to classify variants. All these tools highly depend on existing annotations at corresponding loci. In 2015, Alipanahi et al. [19] developed DeepBind based on convolutional neural networks (CNNs) to predict the binding affinity between TFs and DNA or RNA binding proteins and RNA. DeepSEA [20] applied similar methods to predict the effect of noncoding variants on binding affinity and then classified variants through logistic regression into functional or nonfunctional groups. All tools mentioned above identified expression-modulating variants through indirect inference, because they were not trained on expression-modulating variants or expression-related data. EnsembleExpr [21] used MPRA data to train an ensemble-based model for characterizing expression-modulating variants directly. ExPecto [22] *ab initio* predicted the variants’ effects on gene expression from 40-kb promoter-proximal sequences and then pinpointed expression-modulating variants. However, there is no comprehensive evaluation of these computational tools based on high-quality expression-modulating variants; therefore, it is difficult for users to choose appropriate tools for their tasks.

**Table 1:** Properties of involved computational tools

**Tool**	**Modeling approach**	**Model feature**	**Output**	**Website**	**Refs.**
FunSeq2	Knowledge-based	Evolutionary parameters; ENCODE summaries; PWMs; likely target genes; biological networks; recurrent elements across cancer samples	Cancer driver mutations	http://funseq2.gersteinlab.org/	[14]
CADD	Supervised learning	Evolutionary parameters; ENCODE summaries; population frequencies; transcript information; protein-level scores	Functional variants	https://cadd.gs.washington.edu/	[16]
GWAVA	Supervised learning	Evolutionary parameters; ENCODE summaries; population frequencies	Disease-related variants	https://www.sanger.ac.uk/science/tools/gwava	[17]
Eigen	Unsupervised learning	Evolutionary parameters; ENCODE summaries; population frequencies	Functional variants	http://www.columbia.edu/∼ii2135/eigen.html	[18]
DeepSEA	Supervised learning (DL)	Local sequences; evolutionary parameters	Functional variants	http://deepsea.princeton.edu/	[20]
EnsembleExpr	Ensemble-based	Including features used by DeepSEA, DeepBind, KSM, and ChromHMM	Expression-modulating variants	http://ensembleexpr.csail.mit.edu/	[21], [23], [24]
ExPecto	Supervised learning (DL)	Local sequences	Expression-modulating variants	https://hb.flatironinstitute.org/expecto/	[22]

*Note*: ENCODE, Encyclopedia of DNA Elements; PWM, position weight matrix; DL, deep learning; KSM, *k*-mer set memory.

Here, we present a repository for expression-modulating variants (REVA). The current release of REVA consists of over 11.8 million experimentally validated expression-modulating variants in the human genome, curated with extensive functional annotations. We further benchmark seven popular computational tools in identifying expression-modulating variants [14], [16], [17], [18], [20], [21], [22] based on high-quality data in REVA. All data and benchmarking results are publicly available at http://reva.gao-lab.org.

## Construction and content

### Data collection and integration

**Figure 1:**
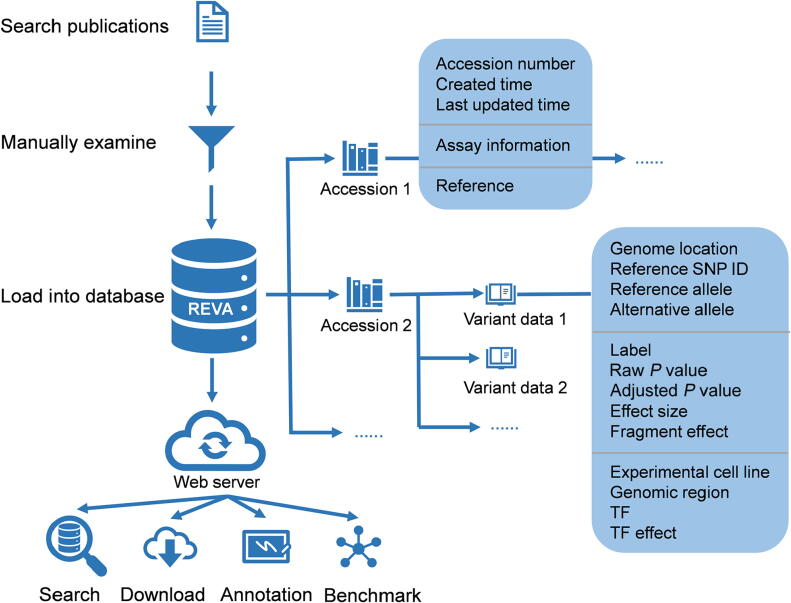
**Overview of the structure of REVA** Manually curated noncoding variant data, as well as supplementary information, were stored in the database at two levels: accession and variant data. Accession contained the information about the publication, and variant data contained all related information about the variant. A web interface was built for users to access the data in the database. TF, transcription factor; SNP, single nucleotide polymorphism.

To ensure unified and high-quality data, all records in REVA were collected and curated using a standard procedure (Figure 1). We used a list of keywords, “MRPA”, “STARR-seq”, “CRE-seq” with “mutation”, “variant”, and “variation”, to retrieve publications from PubMed (https://pubmed.ncbi.nlm.nih.gov/) and then manually checked the abstracts and full texts of the matching publications to obtain literatures that experimentally validated the effects of noncoding expression-modulating variants.

For filtered literatures, we extracted related information of the variants from the main texts as well as supplementary materials of publications and converted them to the same format (Table 2). Variants that failed to be mapped to both GRCh37 and GRCh38 were removed. Variants only mapped to the coding region were also removed. For missing information, we used “.” as a placeholder. In addition, the detailed protocols and raw data of the experiments were also extracted.

**Table 2:** Variant information extracted during the data collection process

**Information**	**Note**
Genome location	Genome location of the variant in both GRCh37 and GRCh38Strand information was also included
Reference SNP ID	Reference SNP ID of the variant
Reference allele	Reference allele of the variant
Alternative allele	Alternative allele of the variant
Raw *P* value	Raw *P* value given by the publication
Adjusted *P* value	If the publication did not provide adjusted *P* value, the method of Benjamini and Hochberg was conducted
Cutoff	The cutoff for the adjusted *P* valueIf the publication did not provide a cutoff, the cutoff was set to 0.05
Label	Given based on the cutoff for the adjusted *P* value provided in the publicationIf the adjusted *P* value was less than the cutoff, the label would be 1; otherwise, the label would be 0
Effect size	Effect size provided by the publication
Fragment effect	The effect of the fragment carrying the variant, given based on the effect size: activation, repression, or no effect
Experimental cell line	The cell line used to conduct the experiment
Genomic region	The genomic region in which the variant was located, such as the particular gene and intron
TF	TF related to the variant
TF effect	The effect of the aforementioned TF: activation or repression

*Note*: SNP, single nucleotide polymorphism; TF, transcription factor.

For variants with the same chromosome, genome location, reference allele, alternative allele, and experimental cell line from different publications were subjected to a meta-analysis to integrate data. The harmonic mean *P* value (HMP) method [25] was used in the meta-analysis, and the cutoff for the meta *P* value was set to 0.001 to generate the meta-label. The variants involved in the meta-analysis but without a raw *P* value were also removed.

The label of variants was given based on the cutoff for the adjusted *P* value or meta *P* value, and then variants were classified into positive variants and negative variants based on label or meta-label. If the variant’s label was 1, the variant was a positive variant and considered to have effects on gene expression; otherwise, it was a negative variant without effect on gene expression.

### Database construction

**Supplementary figure 1:**
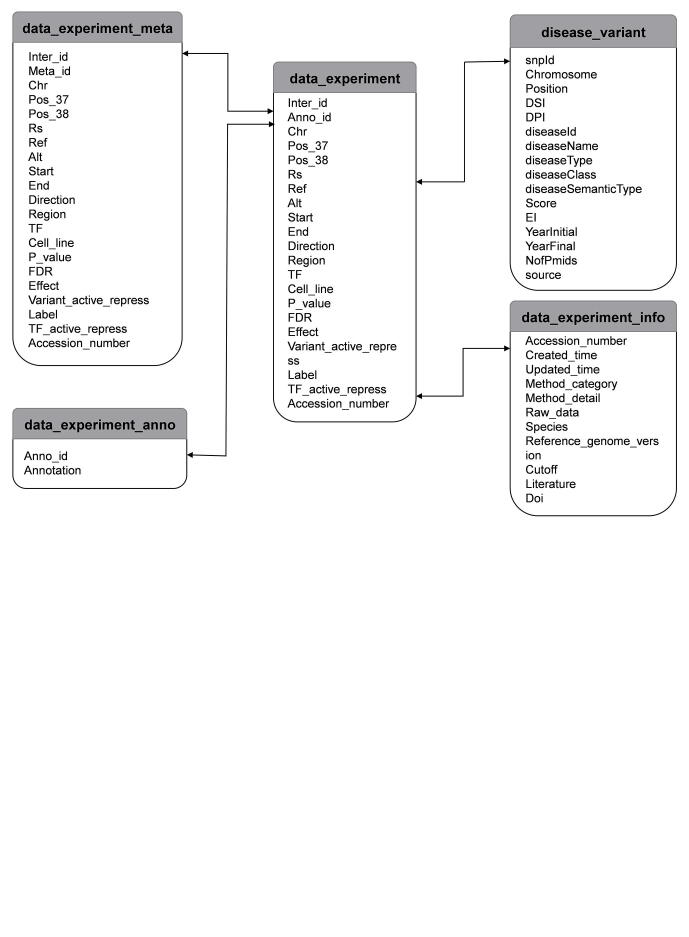
**Database scheme of REVA**. All data were stored in MongoDB with five collections. “data_experiment_info” contained accession information and “data_experiment” contained variant data. The original variant data included in meta-analysis stored in “data_experiment_meta”. “data_experiment_anno” and “disease_variant” contained variant annotation and associated diseases and phenotypes.

All manually curated variant data, as well as meta-information, were stored in MongoDB (https://www.mongodb.com/) at two levels: accession and variant data (Figure S1). Each accession entry consisted of an accession number, created time, last updated time for the accession, information about the assay used in the publication (method type, original reference genome version, link to raw data, and summary of the assay), and the reference. Variant data included all related information about the variant, and each variant data entry was linked to one accession. For the data involved in the meta-analysis, the variant data contained the results of the meta-analysis and were linked to all related variants and accessions.

We also integrated DisGeNET v7.0 (https://www.disgenet.org/) [26] variant-disease associations, GWAS catalog (https://www.ebi.ac.uk/gwas/), ClinVar (https://www.ncbi.nlm.nih.gov/clinvar/), COSMIC (https://cancer.sanger.ac.uk/cosmic), and three-dimensional interacting genes and chromatin state from 3DSNP [27] to our database for providing more variant information.

### Variant annotation

In efforts to pinpoint plausible regulatory mechanisms for these variants, we used 2403 trained CNNs to annotate the functional effects of sequence variations [28] based on 1249 TF binding profiles, 766 histone modification profiles, 280 DNA accessibility profiles, and 108 DNA methylation profiles from the recent Encyclopedia of DNA Elements (ENCODE) data.

NVIDIA Tesla P100 Graphics Processing Units with the implementation on the deep learning framework TensorFlow (https://www.tensorflow.org/) and Python (https://www.python.org/) were used for training models. We adopted stochastic gradient descent (SGD) as the optimizer, and the initial learning rate was 0.01.

The final output layer of the CNN model was a fully connected layer with a sigmoid function used to scale the output between 0 to 1. The input layer was a one-dimensional convolution layer with the thresholded rectified linear unit (ReLU) as the activation function. Next, the max-pooling layer was performed to reduce the complexity of the data. Then, the dropout layer was used to mitigate the overfitting problem. The next two layers were a fully connected layer with thresholded ReLU as the activation function and a dropout layer.

For TF binding, histone modification, and DNA accessibility models, the positive data for training CNNs were the 200-bp sequences centered on the peak in ENCODE profiles. Then we removed positive sequences from the human reference genome and split the rest into 200-bp bins. Random sampled 200-bp bins with the same number of positive data were used as negative data. For DNA methylation, the 200-bp sequences centered on the target base with the methylation rate more than 0.5 or less than 0.5 in whole-genome bisulfite sequencing (WGBS) data were considered as positive data and negative data, respectively. One-hot encoding was conducted to transform each sequence to a 200 × 4 binary matrix for model training.

A five-fold cross-validation strategy was used to train models. During each iteration of model training, 15% of the input data were randomly selected as the independent testing dataset to evaluate model performance. The remaining data were split with 70% to train models and 15% as the validation dataset to optimize parameters. Model performance was evaluated with the area under the receiver operating characteristic curve (AUROC) and area under the precision-recall curve (AUPRC) to test the sensitivity and specificity, and models with the best performance were selected for variant annotation. An average AUROC and an average AUPRC of 2403 models were reported.

To character the binding affinity changes of the variant, we used 2403 trained CNNs to predict on 200-bp sequences centered with the reference allele and alternative allele, respectively. For each chromatin profile, the log_2_ fold change (as the method shown in DeepSEA) [20] was calculated as the variant effect on chromatin profile. Specifically,

$$Effect={log}_{2}\left(\frac{P_{r}}{1-P_{r}}\right)-{log}_{2}\left(\frac{P_{a}}{1-P_{a}}\right)$$

where $P_{r}$ was the prediction of sequence with reference allele, and $P_{a}$ was the prediction of sequence with alternative allele.

Furthermore, we incorporated 13 DNA physicochemical properties and 8 evolutionary features into the annotation pipeline. The 13 physicochemical properties were calculated as described by Li et al. [29], and 8 conservation scores were downloaded from UCSC Genome Browser (http://genome.ucsc.edu/).

### Benchmarking

To prepare the benchmarking dataset for evaluating the performance of state-of-the-art computational tools in calling expression-modulating variants based on the curated data in REVA, we first excluded loci tested in mice (*n* = 15,152). There are overlapping variants between the training datasets of state-of-the-art tools and the REVA benchmarking dataset. If the benchmarking dataset contains these variants, the performance of related tools will be overestimated. To avoid the influence of these variants and make a fair comparison, we further removed variants (*n* = 47,518) that were either found in the GWAVA [17] and EnsembleExpr [21] training datasets or used to compute the empirical background distributions by DeepSEA [20]. For the remaining 5,809,991 loci (37,816 positive and 5,772,175 negative), we ran CADD (v1.4, https://cadd.gs.washington.edu/download) [16], DeepSEA (http://deepsea.princeton.edu/), EnsembleExpr (https://github.com/gifford-lab/EnsembleExpr/), and ExPecto (https://hb.flatironinstitute.org/expecto/) [22], and used precomputed score sets of Eigen (v1.1, http://www.columbia.edu/~ii2135/download.html) [18], FunSeq2 (v2.1.6, http://funseq2.gersteinlab.org/downloads) [14], and GWAVA (https://www.sanger.ac.uk/science/tools/gwava), to obtain the corresponding predicted score for evaluation. The thresholds used in the evaluation were those recommended by the corresponding papers or official websites (Table S1).

All variants in the benchmarking dataset were variants with expression-modulating potential. One of the biological mechanisms by which disease-related or phenotype-related variants function is having effects on gene expression regulation [30]. Pinpointing disease-related or phenotype-related variants is more useful for biomedical researches. Therefore, we further selected the GWAS, ClinVar, and HGMD subsets of the benchmarking dataset to test these tools’ power.

## Results

### Characterization and distribution of expression-modulating variants

**Figure 2:**
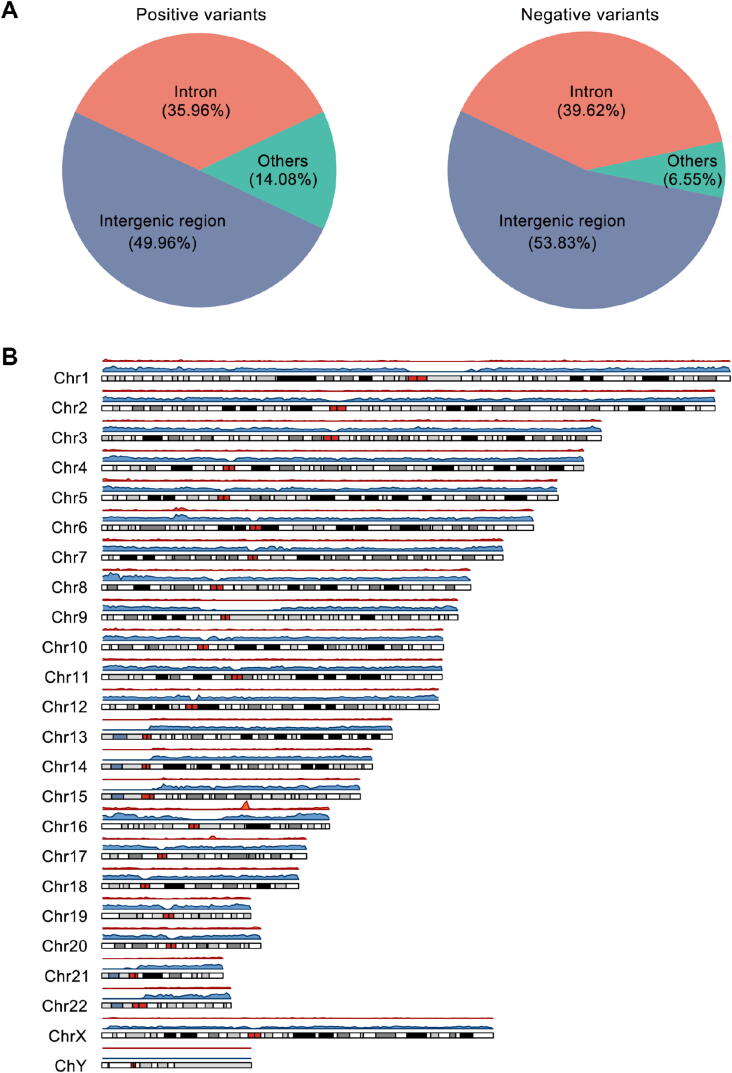
**Annotation of the variants in REVA A.** Distribution of positive and negative variants in human genome. **B.** Density distribution of positive and negative variants on chromosomes. A two-sided Fisher's exact test with Benjamini and Hochberg correction [42] was used in the analysis of the chromosome distribution of variants. The cutoff for the adjusted *P* value was set to 0.05. The density distribution plot was constructed with the karyoploteR package [43] in R. No variants were located on the Y chromosome.

**Supplementary figure 2:**
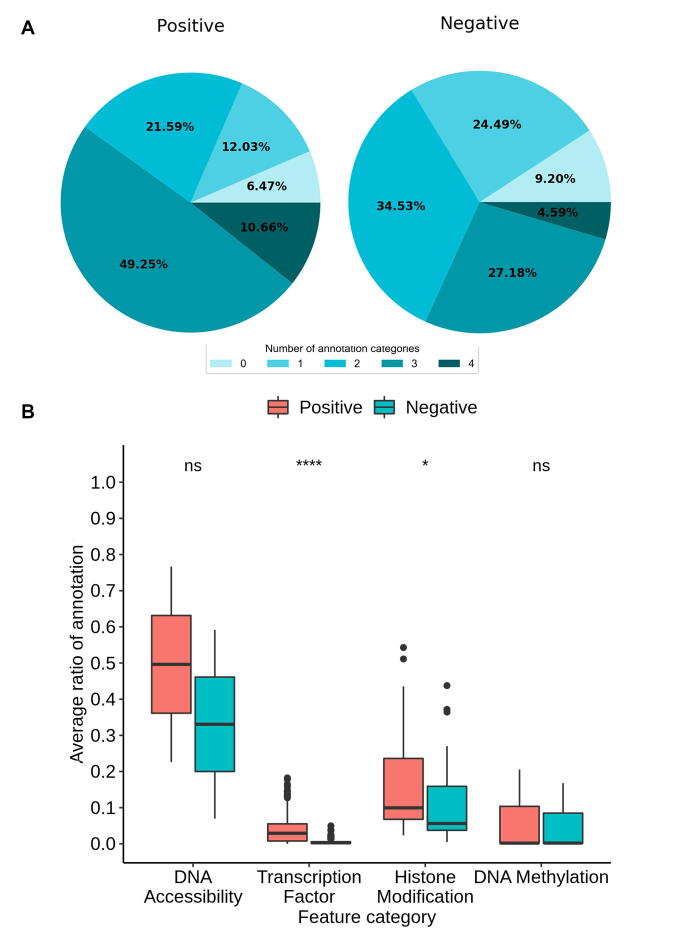
**Annotation of the variants in REVA**. A. The ratio of positive and negative variants that have 0, 1, 2, 3, or 4 categories of annotations. B. The average ratio of annotations of variants by category.

All curated expression-modulating variants were validated by experiments, and we applied standard data collection and integration procedure to ensure the high-quality data with the unified format. By the end of November 2019, REVA consisted of 11,862,367 entries covering 5,948,789 experimentally tested noncoding loci across 18 cell cultures from 14 publications [4], [8], [11], [31], [32], [33], [34], [35], [36], [37], [38], [39], [40], [41]. We first excluded loci tested in mice (*n* = 15,152) and with more than one alternative allele (*n* = 26,276). Among the remaining 5,907,361 loci (34,700 positive and 5,872,661 negative), most were located in intergenic (positive: 49.96%, negative: 53.83%) and intronic (positive: 35.96%, negative: 39.62%) regions (Figure 2**A**; Table S2). We found that both positive and negative variants were unevenly distributed on chromosomes, and no variants were located on the Y chromosome (Figure 2B; Table S3). Specifically, fewer positive variants were located on chromosomes 1, 3, 5, 13–15, and 21, and the X chromosome, and more positive variants were located on chromosomes 6, 8, 10–12, and 16–20. Fewer negative variants were located on chromosomes 9, 13–15, 21, and 22, and the X chromosome, and more negative variants were located on chromosomes 1–8, 10–12, and 16–20. Biochemical activities were detected for 93.53% positive and 90.80% negative cases in at least one cell culture (Figure S2A; Tables S4 and S5). Of note, more positive than negative variants were found in TF binding regions, highlighting the contribution of TF binding changes to expression modulation (Figure S2B).

### Extensive functional annotation of expression-modulating variants

We used 2403 trained CNNs to annotate the functional effects of expression-modulating variants [28]. Most of the trained CNNs were accurate, with an average AUROC of 0.908 and an average AUPRC of 0.904. Among the 5,789,688 variants annotated, both positive and negative variants were found to lead to significant changes in binding affinity for 22 and 12 TFs on average, respectively, which also suggested that expression-modulating variants may affect gene expression regulation through changing the binding affinity of TFs. Moreover, 8.72% positive and 3.56% negative variants were located at evolutionary conserved loci (phastCons100way score > 0.6).

### Benchmarking of state-of-the-art computational tools

To evaluate the power of state-of-the-art computational tools in calling expression-modulating variants, we further benchmarked multiple state-of-the-art computational tools based on the curated data in REVA. With the benchmarking dataset containing 5,809,991 loci (37,816 positive and 5,772,175 negative), we found that 1289 could not be predicted by DeepSEA (since their evolutionary features were not available), and 560,577 were not included in the precomputed score set of Eigen, FunSeq2, and GWAVA, so we further excluded these 561,866 cases from follow-up analysis. Meanwhile, as EnsembleExpr could not finish the whole benchmarking dataset in a reasonable amount of time, we assessed its performance based on the average metrics over 5 randomly sampled sub-datasets with 368 positive and 56,026 negative cases on average.

**Figure 3:**
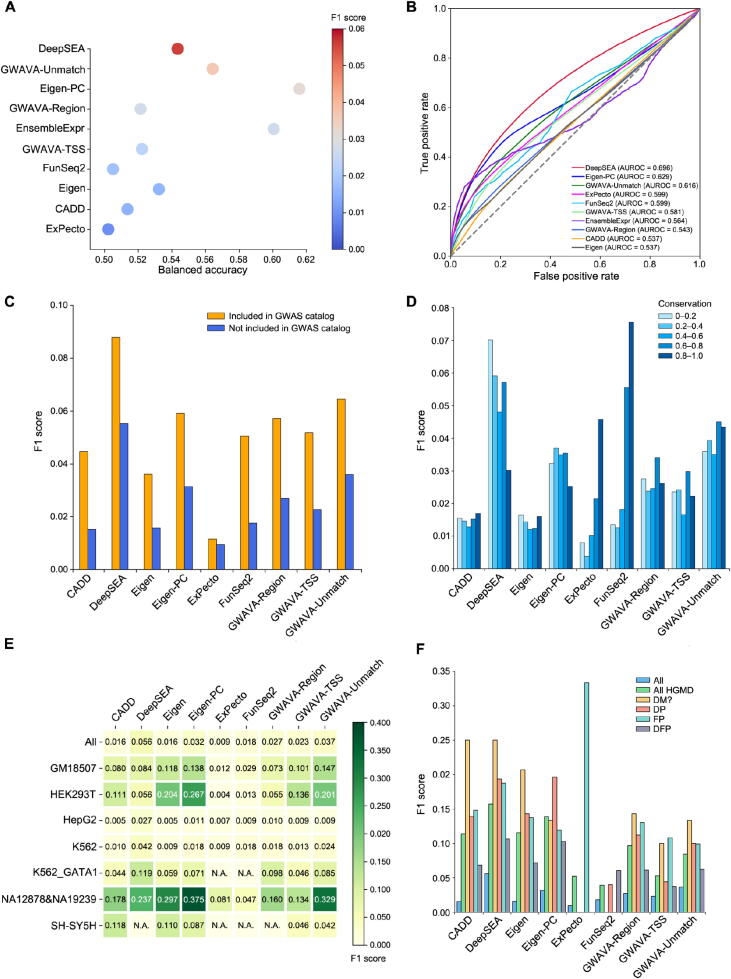
**Performance of involved tools on the benchmarking dataset A.** Performance comparison of involved tools. Bubbles are colored by F1 scores. The tools are ordered by F1 scores. **B.** The ROC curves for involved tools. **C.** Performance comparison of involved tools except for EnsembleExpr on variants that were also included in GWAS catalog. **D.** Performance comparison of involved tools except for EnsembleExpr on variants with different phastCons100way scores. **E.** Performance comparison of involved tools except for EnsembleExpr on variants from different cell lines. “All” represents the F1 score shown in (A). **F.** Performance comparison of involved tools except for EnsembleExpr on variants that were also included in HGMD. “All” represents the F1 score shown in (A). “All HGMD” represents the F1 score on all variants that were also included in HGMD. “DM?”, “DP”, “FP”, and “DFP” refer to the classes of related variants documented in HGMD. AUROC, area under the receiver operating characteristic curve; N.A., not available.

Overall, the best-performing tool was DeepSEA, with the highest AUROC and F1 score (Figure 3**A and B**; Table S6). All tools performed well in terms of specificity but poorly in terms of sensitivity. EnsembleExpr had the highest sensitivity but the lowest specificity, whereas ExPecto showed the best specificity and worst sensitivity (Table S6).

There were 52,672 variants in the benchmarking dataset that overlapped with the GWAS catalog (v1.0.2), and 658 of them were positive variants. All tools performed better on variants overlapping with the GWAS catalog, and DeepSEA still had the best performance (Figure 3C). Meanwhile, ExPecto and FunSeq2 showed better performance on variants at evolutionarily conserved loci, while DeepSEA displayed moderate performance (Figure 3D).

The coverage and quality of training data may contribute significantly to the performance of machine learning-based models [44]. To test whether variants from different cell lines would affect the performance of these tools, we further evaluated these tools separately on seven cell lines (Table S7). On GM18507, GWAVA-Unmatch performed best; on HEK293T and NA12878&NA19239, Eigen-PC had the highest F1 score; DeepSEA had the best performance on HepG2, K562, and K562_GATA1; and CADD performed best on SH-SY5H (Figure 3E), which suggested that the diversity of the original training data contributes to the performance differences of these tools. Of note, thus far, only ExPecto outputted cell type-specific scores for various tissues.

**Supplementary figure 3:**
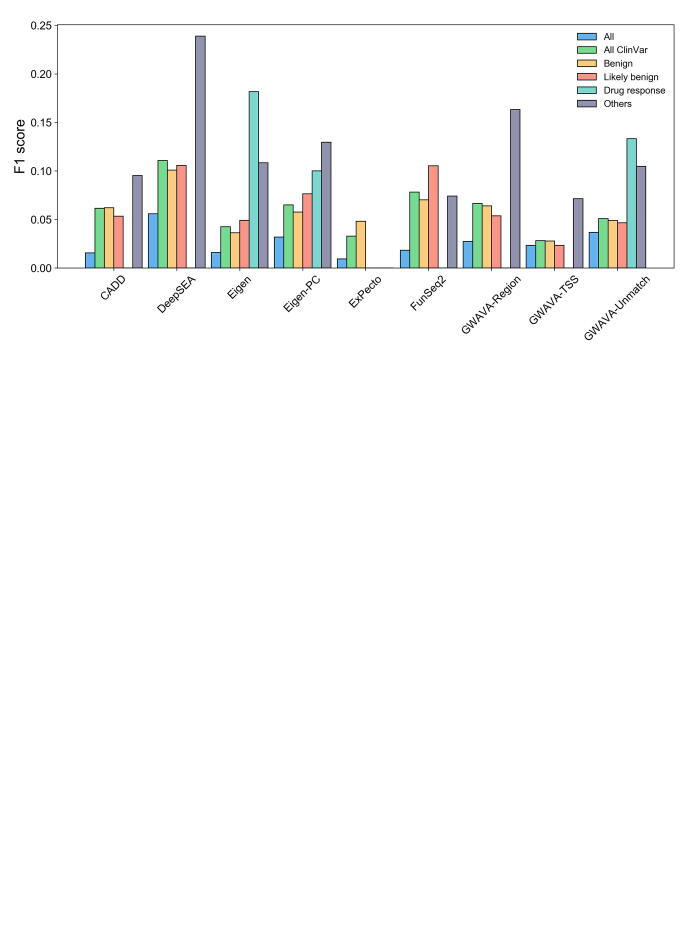
**Performance comparison of involved tools except for EnsembleExpr on variants that were also included in ClinVar**. “All” represents the F1 score in Figure 3A. “All ClinVar” represents the F1 score on all variants that were also included in ClinVar. “Benign”, “Likely benign”, and “Drug response” refer to the clinical significance of related variants documented in ClinVar.

To provide a further explanation of the potential mechanisms of disease-related variants, we evaluated the benchmarking dataset on disease-related variants. There were 1400 variants in the benchmarking dataset that overlapped with HGMD (2019.3 professional), and 69 of them were positive variants (Table S8). Moreover, 8 of 69 variants were verified to regulate gene expression by independent experiments; 40 of 69 variants were associated with diseases such as colorectal cancer, nervous system diseases, and autoimmune diseases. To test computational tools’ power on disease-related variants, we compared their performance on these variants. All tools performed better on variants overlapping with HGMD, and DeepSEA still had the best overall performance (Figure 3F), same on variants with class “DM?” and “DFP”. Eigen-PC showed the best performance on variants with class “DP”. Interestingly, ExPecto performed best on variants with class “FP” but worst on variants with other classes. We also evaluated variants overlapping with ClinVar (2019.10.08), and DeepSEA had the best overall performance, and Eigen showed the best performance on “Drug response” related variants (Figure S3; Table S9).

### Web interface

**Figure 4:**
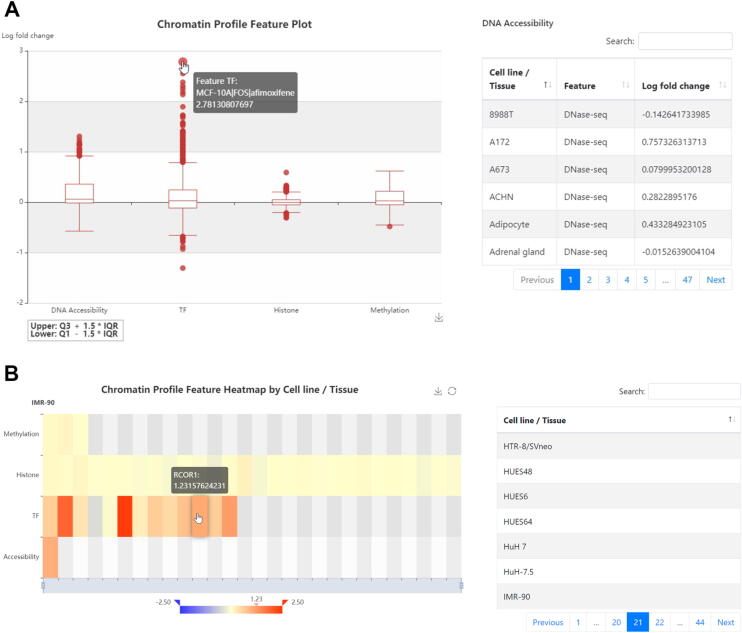
**Illustration of the Web interface of REVA A.** Chromatin profile feature plot in “Annotation” module of the variant detail page. Chromatin features are presented by category. Users can hover the mouse over the outlier or the box to show more information. At the right of the boxplot is a table to show detailed information. Users can click the boxplot to show the corresponding category. **B.** Chromatin profile feature heatmap in “Annotation” module of the variant detail page. The heatmap is presented by cell line and each row in heatmap corresponding to one category. Users can click the “Cell line / Tissue” list at the right of the heatmap to render annotation in the target cell line / tissue and hover the mouse over the block in the heatmap to show feature information. Both (A) and (B) were retrieved from http://reva.gao-lab.org/detail.php?id=intid1_8498680_8438620a.tk562&reference=GRCh38. IQR, interquartile range.

**Supplementary figure 4:**
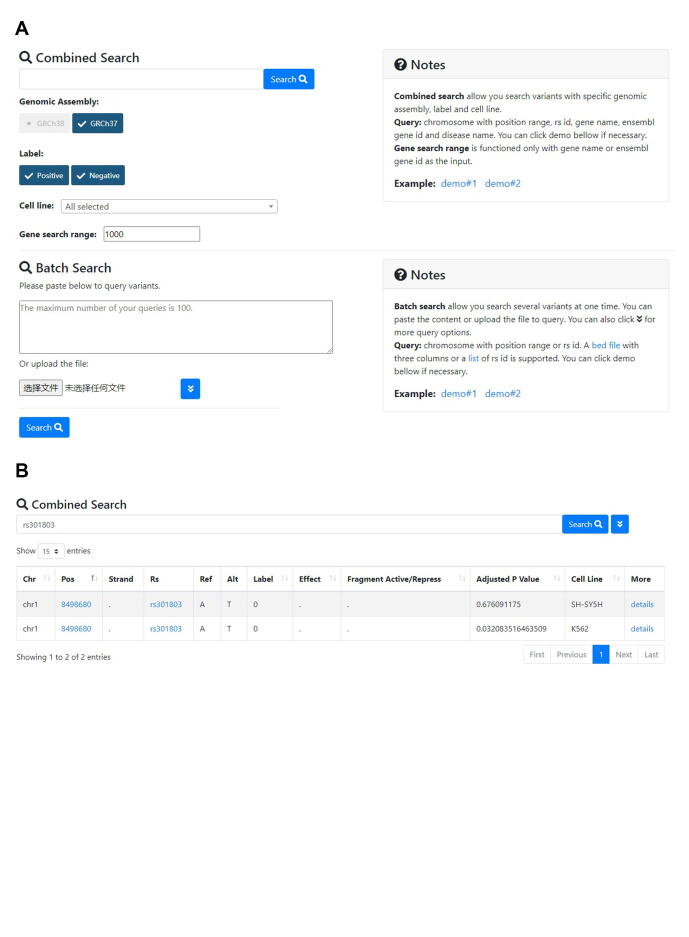
**Illustration of the web interface of REVA**. A. Advanced search page (http://reva.gao-lab.org/advanced_search.php). Users can conduct customized searches or batch searches through this page. B. Search results page. Users can sort searching results by “Chr”, “Pos”, “Label”, “Effect”, “Fragment Active/Repress”, “Adjusted P Value”, or “Cell Line”.

REVA (http://reva.gao-lab.org) provides an interactive web interface for users to explore all data entries and analysis results (Figure 4, Figure S4). Users can start a quick search by chromosome position, rs ID, gene name, ensemble gene ID, or disease name. “Advanced search” provides a customized search and batch search for users. The query result is presented as a table, which includes basic information, expression information (such as the label, effect size, and adjusted *P* value), and the related genomic region. Users can directly click the link of position and rs ID to access UCSC Genome Browser and dbSNP (https://www.ncbi.nlm.nih.gov/snp/) for more information. Users can also click the “details” link for more information. The detail page contains eight modules: “Basic Information”, “Cell Line and Expression”, “Three-dimensional Interacting Gene”, “Chromatin State”, “Disease and Phenotype”, “Meta Sources” (only available for variants involved in meta-analysis), “Accession”, and “Annotation”. In the “Annotation” module, chromatin profile features are rendered as a heatmap by cell line and a boxplot by category, and DNA physicochemical properties and evolutionary features are presented as responsive tables. Users can download the annotation for further analysis. Moreover, we also provide benchmarking results of state-of-the-art computational tools. Users can download all variants in REVA and the benchmarking dataset through the “Download” page.

### Explore plausible regulatory mechanisms of expression-modulating variants

Autoimmune diseases are caused by the abnormal immune response to attack and damage functional tissues due to complex interactions between environmental and genetic factors [45]. GWAS and fine-mapping studies have identified thousands of noncoding variants associated with autoimmune diseases [46]. Since the mechanisms of autoimmune disease are complicated, pinpointing causal variants and exploring their possible functional mechanisms remain a challenge [47].

**Supplementary figure 5:**
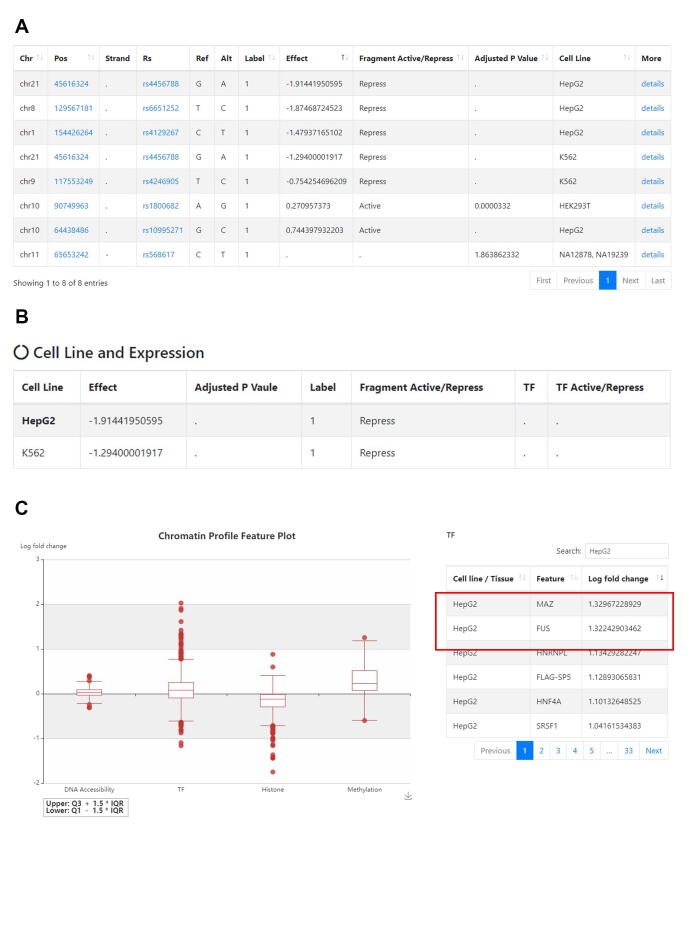
**The combined search result of “ankylosing spondylitis”**. A. Searching result sorted by “Effect”. Variant rs4456788 has the largest effect size in HepG2 cell line. B. “Cell Line and Expression” module in the variant detail page of rs4456788. This variant is also considered to repress gene expression in K562 cell line. C. The chromatin profile feature plot of rs4456788 focusing on transcription factor binding in HepG2 cell line, sorted by log2-fold-changes. The variant is considered to decrease the binding affinity of transcription factors MAZ and FUS.

Ankylosing spondylitis is a kind of chronic autoimmune disease, but the pathogenesis remains unclear [48]. On the advanced search page of REVA (Figure S5), we filtered the label to positive and searched with “ankylosing spondylitis”. The search result contained 8 entries, and among them, the variant rs4456788 (near the *ICOSLG* locus) had the largest effect size tested in HepG2 cell line and was considered to repress expression. It was also tested in K562 cell line and resulted in the same conclusion. Through the annotation module of the detail page, we found that in HepG2 cell line, the alternative allele of rs4456788 could decrease the binding affinity of TFs MAZ and FUS. MAZ has been proven to have bidirectional transcriptional regulation [49], and FUS has a transcriptional activation function [50]. It could be the possible regulatory mechanism of rs445678, and this might be helpful for further researches on the mechanism of pathogenesis of ankylosing spondylitis.

## Discussion

REVA is a database specifically designed for storing experimentally validated expression-modulating data. It currently consists of 11,862,367 entries covering 5,948,789 experimentally tested noncoding loci across 18 cell cultures. Both experimentally validated expression-modulating variants and meta-information about assays were curated. Comparing with the existing database, REVA is the largest database designed for curating experimentally validated expression-modulating noncoding variants specially. Besides, we provide 2424 functional annotations, including TF binding, epigenetic modifications, DNA accessibility, DNA physicochemical properties, and evolutionary features.

Most of the variants in REVA were located in intergenic and intronic regions and were unevenly distributed on chromosomes. Several factors may contribute to the uneven distribution. First, it has been well demonstrated that the functional elements are unevenly distributed across chromosomes [51], [52]. Consistently, we found that the numbers of both positive and negative variants were highly correlated with the gene numbers across all chromosomes (Pearson’s *r* = 0.80, *P* = 2.6 × 10^−6^ for positive variants; Pearson’s *r* = 0.82, *P* = 7.1 × 10^−7^ for negative variants). Moreover, technical challenges counted too. In particular, the Y chromosome had long been taken as a “genetic wasteland” [53] and excluded from genomic analyses for quite some time due to its genetic and structure complexities [54]. Although this idea has been shifted with more researches on chromosome Y, the underrepresentation of chromosome Y on commonly used arrays still exists [55]. We also noticed that certain experimental designs may lead to reporting bias [8], [33], [38], [39], [40], [41]. However, after removing data generated from studies designed for assessing particular regions [38] or elements [8], [33], [39], [40], [41], we found that the uneven distribution remains.

Furthermore, we provide a high-quality benchmarking dataset for evaluating state-of-the-art computational tools designed for identifying expression-modulating variants as well as benchmarking results of multiple published computational tools as a reference for users to select the best tools for their particular tasks. Overall, all seven tools have high specificity but low sensitivity. DeepSEA has the best performance on the whole benchmarking dataset in terms of AUROC and F1 score, and all tools have better performance on disease-related or phenotype-related variants, suggesting that the diversity of the original training data of these tools contributes to different performance across different benchmark subsets. We noticed that not all tools involved in the benchmark were designed for identifying expression-modulating variants originally, and a “negative” expression-modulating noncoding variant might also be associated with disease via non-transcription mechanisms like epigenetic marks [56] or chromatin structuration [57].

**Supplementary figure 6:**
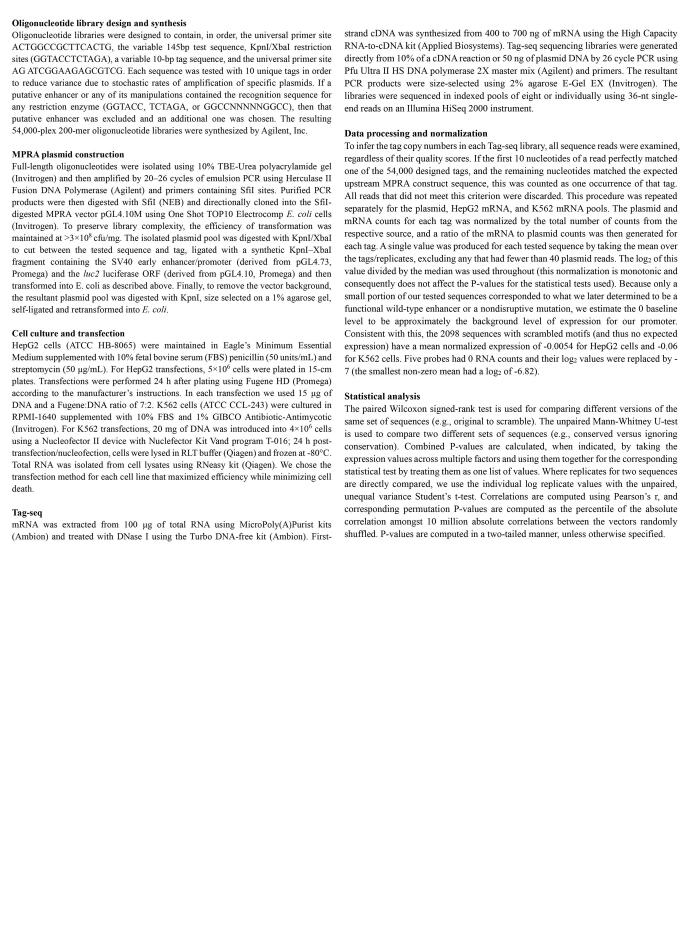
**Illustration of the protocol details documented in REVA**. Protocol details were extracted from the original literature and as an attachment in the accession (http://reva.gao-lab.org/download/protocol/reva-0002.pdf).

It should be noted that not all variants collected in our database were tested by identical experimental protocols. Non-saturation mutagenesis-based studies examine several elements at a time, and each fragment usually contains one variant, with the effect size calculated by counting reads directly [8] or employing a linear model [32]. Meanwhile, saturation mutagenesis-based studies focus on a few elements; each fragment contains two or more variants, and the effect size is calculated through linear regression [41]. Protocol details for each variant were documented during curation to help users interpret records effectively (Figure S6).

We believe that this database will be useful for not only computational but also bench biologists in genomics, bioinformatics, and genetics communities, and we will keep the resources updated with new data and annotations that emerge in the coming years.

Data availability

REVA is freely accessible at http://reva.gao-lab.org.

Code availability

Source code for all analyses and benchmarking is available on GitHub at https://github.com/gao-lab/REVA-Data_Source_Code.

Competing interests

The authors declare no competing interests.

### CRediT authorship contribution statement

**Yu Wang:** Methodology, Software, Data curation, Formal analysis, Visualization, Writing – original draft, Writing – review & editing. **Fang-Yuan Shi:** Methodology, Software, Data curation, Formal analysis. **Yu Liang:** Data curation. **Ge Gao:** Conceptualization, Project administration, Supervision, Funding acquisition, Resources, Writing – review & editing.
